# Examining the Effect of Increased Aerobic Exercise in Moderately Fit Adults on Psychological State and Cognitive Function

**DOI:** 10.3389/fnhum.2022.833149

**Published:** 2022-07-12

**Authors:** Julia C. Basso, Douglas J. Oberlin, Medha K. Satyal, Catherine E. O’Brien, Christen Crosta, Zach Psaras, Anvitha Metpally, Wendy A. Suzuki

**Affiliations:** ^1^Department of Human Nutrition, Foods, and Exercise, Virginia Tech, VA, United States; ^2^School of Neuroscience, Virginia Tech, VA, United States; ^3^Center for Health Behaviors Research, Fralin Biomedical Research Institute at VTC, Roanoke, VA, United States; ^4^Center for Neural Science, New York University, New York, NY, United States; ^5^Department of Health Sciences, Lehman College, City University of New York, Bronx, NY, United States

**Keywords:** physical activity, cardiopulmonary fitness, spatial learning and memory, episodic memory, mood, affective state, body image

## Abstract

Regular physical exercise can decrease the risk for obesity, diabetes, and cardiovascular disease, increase life expectancy, and promote psychological health and neurocognitive functioning. Cross-sectional studies show that cardiorespiratory fitness level (VO_2_ max) is associated with enhanced brain health, including improved mood state and heightened cognitive performance. Interventional studies are consistent with these cross-sectional studies, but most have focused on low-fit populations. Few such studies have asked if increasing levels of physical activity in moderately fit people can significantly enhance mood, motivation, and cognition. Therefore, the current study investigated the effects of increasing aerobic exercise in moderately fit individuals on psychological state and cognitive performance. We randomly assigned moderately fit healthy adults, 25–59 years of age, who were engaged in one or two aerobic exercise sessions per week to either maintain their exercise regimen (*n* = 41) or increase their exercise regimen (i.e., 4–7 aerobic workouts per week; *n* = 39) for a duration of 3 months. Both before and after the intervention, we assessed aerobic capacity using a modified cardiorespiratory fitness test, and hippocampal functioning *via* various neuropsychological assessments including a spatial navigation task and the Mnemonic Similarity Task as well as self-reported measures including the Positive and Negative Affect Scale, Beck Anxiety Inventory, State-Trait Anxiety Inventory, Perceived Stress Scale, Rumination Scale, Eating Disorders Examination, Eating Attitudes Test, Body Attitudes Test, and Behavioral Regulation of Exercise Questionnaire. Consistent with our initial working hypotheses, we found that increasing exercise significantly decreased measures of negative affect, including fear, sadness, guilt, and hostility, as well as improved body image. Further, we found that the total number of workouts was significantly associated with improved spatial navigation abilities and body image as well as reduced anxiety, general negative affect, fear, sadness, hostility, rumination, and disordered eating. In addition, increases in fitness levels were significantly associated with improved episodic memory and exercise motivation as well as decreased stress and disordered eating. Our findings are some of the first to indicate that in middle-aged moderately-fit adults, continuing to increase exercise levels in an already ongoing fitness regimen is associated with additional benefits for both psychological and cognitive health.

## Introduction

Maintaining a regular exercise regimen is an important health behavior to control weight, strengthen muscles and bones, increase flexibility, decrease risk for diabetes, obesity, heart disease, arthritis, and cancer, and increase life expectancy (Penedo and Dahn, 2005; Wen et al., 2011). In addition, exercise aids in psychological and neurological health, improving affective state and cognitive functioning as well as delaying the onset of brain atrophy and neurodegenerative disorders (Hillman et al., 2008; Hearing et al., 2016). Importantly, individuals who remain more physically active during adulthood have a lower risk for cognitive decline, mild cognitive impairment, dementia, or Alzheimer’s disease during the aging process (Yaffe et al., 2001; Abbott et al., 2004; van Gelder et al., 2004; Weuve et al., 2004; Taaffe et al., 2008; Hamer and Chida, 2009; Geda et al., 2010; Kirk-Sanchez and McGough, 2014; Hörder et al., 2018). This effect appears to follow a dose-response curve, with those individuals exercising at the highest levels showing the greatest neuroprotective effect (Yaffe et al., 2001; van Gelder et al., 2004; Hamer and Chida, 2009; Erickson et al., 2010). However, the 2018 Physical Activity Guidelines Advisory Committee Report indicates that, “insufficient evidence is available to determine whether a dose-response relationship exists between physical activity and cognition because of conflicting findings across populations, cognitive outcomes, and experimental approaches,” indicating that this an important area of research (Piercy et al., 2018).

In the human literature, the most prominent effects of both acute and chronic exercise have been demonstrated in mood and executive functions dependent on the prefrontal cortex, such as attention, working memory, cognitive flexibility, and inhibitory control (Chang et al., 2012; Basso and Suzuki, 2017; Loprinzi et al., 2021). Recent findings have also shown evidence of exercise-induced improvements in hippocampal-dependent function including high-interference memory (Déry et al., 2013; Heisz et al., 2017; Suwabe et al., 2017a; Bernstein and McNally, 2019) and recognition memory (Whiteman et al., 2014). In line with these findings, the primary effects of exercise in the rodent literature have shown significant effects on the hippocampus including structural changes such as neurogenesis, synaptogenesis, gliogenesis, and increased volumetric growth leading to physiological changes that result in lower thresholds of excitation for this system (Pereira et al., 2007; Voss et al., 2013, 2019). Additionally, cross-sectional neuroimaging studies in humans indicate that cardiorespiratory fitness (VO_2_ max) is associated with larger brain volumes of the hippocampus (Erickson et al., 2009) and functional connectivity in the default mode network during resting state analysis, specifically in areas including the parahippocampal gyrus and middle temporal gyri (Voss et al., 2010). At a behavioral level, cross-sectional studies in humans have found an association between physical activity and enhanced hippocampal-dependent function (Cox et al., 2016; Gaertner et al., 2018); however, little has been done using an interventional approach to examine the effects of long-term exercise on hippocampal behaviors, including functions of both the posterior (e.g., spatial learning and memory) and anterior hippocampus (e.g., emotional and motivational behaviors).

Most studies examining the effects of long-term exercise on brain health have been conducted in either children or low fit, elderly populations. Few studies have examined the effects of exercising on psychological state and cognitive health in healthy, moderately fit adults (but see Chen et al., 2019; Quinlan et al., 2021). In addition, many longitudinal exercise studies examining cognitive functioning are not always focused on increasing cardiorespiratory fitness (VO_2_ max) levels (Smiley-Oyen et al., 2008; Ruscheweyh et al., 2011). We chose this population as we hypothesized that young and middle-aged adults will have the physical capabilities to exercise at intensities that will increase VO_2_ max and that exercise may continue to have brain benefits in individuals whose brain health and plasticity are still at heightened levels; that is, before the onset of aging and neurodegeneration. Additionally, we hypothesized that this population with an already ongoing fitness regimen would be particularly motivated to increase their weekly physical activity, thus adhering to the experimental intervention.

To address our hypotheses, we collaborated with an established exercise facility that offered in-person cycling classes to ensure that all participants engaged in a quantifiable and identical (both in terms of mode and duration) aerobic exercise experience. We randomly assigned healthy adults, 25–59 years of age, who were currently engaging in one or two aerobic exercise sessions per week to either maintain their exercise regimen (i.e., one or two indoor cycling sessions per week) or increase their exercise regimen (i.e., 4–7 indoor cycling sessions per week) for a duration of 3 months. Before and after the intervention, we assessed aerobic capacity *via* a modified cardiorespiratory fitness test, cognitive functioning *via* a battery of neurocognitive assessments (i.e., Stroop Task; Eriksen Flanker Task; N-Back Task; Spatial Navigation Task; Mnemonic Similarity Task), and psychological state *via* a series of self-reported questionnaires. We focused on affective state, exercise motivation, eating attitudes, and body image as these psychological processes have been associated with adaptive health behaviors. We hypothesized that participants who increased their exercise regimen would show significant psychological and cognitive improvements over those who maintained their exercise regimen and that these changes would be predicted by increases in cardiorespiratory fitness. Further, we hypothesized that there would be a significant relationship between the workout regimen and the psychological and cognitive changes, such that those who engaged in more exercise sessions or gained the most in terms of fitness over the course of 3 months would show the greatest benefits.

## Methods

### Participants

A total of *n* = 130 participants were recruited from Austin, TX through online and flyer advertisements. All participants were healthy males and females between the ages of 25–59 years of age, with English as their primary language, and who had a moderate and regular exercise regimen (defined as exercising one or two times per week for 20 min or more for the past 3 months). Participants were excluded if they currently smoked, had back, hip or knee issues or other preexisting health conditions that made exercise difficult or unsafe. Smokers were excluded as smoking is an independent risk factor for a variety of chronic conditions, and smoking can impair cardiorespiratory functioning, making exercise difficult or uncomfortable (Johannsen et al., 2014). Participants were also excluded if they had a current diagnosis of and/or took medication for psychiatric or neurological conditions including anxiety, depression, bipolar disorder, schizophrenia, or epilepsy. Prior to participation, all participants gave their informed consent. All study documentation and data collection methods were approved by and in compliance with the New York University Committee on Activities Involving Human Subjects. For the final analysis, participants were excluded if they did not complete 12 or more total workouts (i.e., at least one per week). The final analysis was conducted on a total of *n* = 80 participants; however, some analyses include fewer than *n* = 80 due to missing data. We note in the results where this is the case.

### General Procedures

All participants engaged in 3 months of a physically active experience. Participants were randomly assigned to either maintain their current exercise regimen (control group) or increase their exercise regimen to 4–7 classes per week (experimental group). Control participants were assigned to take either one or two classes based on their previous exercise regimen, which was evaluated *via* a self-reported, screening questionnaire. On the screening questionnaire, participants were asked during the past 3 months, whether exercise was a routine part of their life, how many days per week they exercised, and the level of intensity of these workouts. They were also asked to write a qualitative description of their workout habits. Based on this information as well as follow-up phone calls, if necessary, research personnel determined whether participants were moderately active. Fitness level was later quantified at fitness testing. Experimental participants were encouraged to engage in a minimum of four sessions per week but could increase their exercise regimen to seven per week. All exercise sessions took place at RIDE Indoor Cycling^1^ in Austin, TX. All classes were cycling classes that were 45 min in duration. Participants were given a Mio FUSE^2^, wrist-based heart rate monitor, to wear during all exercise sessions. Heart rate (HR) was captured for each exercise session throughout the course of the 3 months, and participants manually recorded their average HR for each exercise session using MyFitnessPal. Participants self-reported other exercise sessions that occurred outside of RIDE.

Before and after the 3-month period, participants completed a modified cardiorespiratory fitness test as well as a series of neuropsychological tasks and self-reported metrics.

### Fitness Tests

At both the beginning and end of the intervention, a submaximal cycle test was used to assess each participant’s aerobic fitness. Participants placed a heart rate monitor (Polar) around their chest and mounted a stationary bicycle. Because of the remote nature of the study, all fitness assessments were conducted *via* video conferencing (Skype) and remote access to the computer. During the fitness assessment, participants were instructed to maintain a cycle cadence of 50 rotations per minute (RPM). The first 2 min of the fitness assessment was a warm-up period, with no added resistance on the stationary bike. Following the warm-up, the resistance was increased by 6 watts (equivalent to 0.12 kp or 36 kgm/min) every min, with RPE and heart rate being recorded at the end of every min. In addition, RacerMate was attached to the stationary bicycle and tracked speed [both miles per hour (mph) and RPM], watts, and caloric expenditure. Perceived exertion was also monitored each min of the test using the Borg Rating of Perceived Exertion Scale (RPE). The test was continued until at least 3 HRs were recorded between 110 bpm and 80% of age-predicted maximal HR [206 − (0.67 × age)]. The test was terminated when the participant requested to stop, could not maintain an RPM of 50, or they reached or exceeded 80% of their age-predicted maximal HR. Following the fitness assessment, the participants were given a 2-min cool-down period. The maximal aerobic capacity was then estimated by extrapolating the HR response to each workload to an age-predicted maximal HR.

### Calculations

The fitness test was designed to measure several (at least three) workloads and HR responses between 110 BPM and approximately 80% of age predicted maximal HR, also described in the American College of Sports Medicine’s Guidelines for Exercise Testing and Prescription section on sub-maximal cardiorespiratory fitness testing using cycle-ergometers. The HRs were measured in beats per minute and the workloads were recorded as kgm/min. The correlation of these values was determined using the following equation: $\sum \left(x-\underline{x}\right)\left(y-\underline{y}\right)÷\sqrt{\sum {\left(x-\underline{x}\right)}^{2}\sum {\left(y-\underline{y}\right)}^{2}}$, where x values are workloads, and y values are HR’s. This value was then multiplied by the quotient of the standard deviations for each set of points (i.e., $\left(\sqrt{\sum {\left(x-\underline{x}\right)}^{2}÷\left(n-1\right)}\right)÷\left(\sqrt{\sum {\left(y-\underline{y}\right)}^{2}÷\left(n-1\right)}\right)$ to calculate the slope of a best fit line. Then the intercept of the best fit line was calculated as follows: *x − (calculated slope × y*). Thus, with an equation that best describes the HR response to workload, the workload expected to elicit age predicted maximal HR was calculated [*(calculated slope × age predicted max HR) + calculated intercept*]. To estimate the metabolic cost of this predicted maximal workload, the American College of Sports Medicine’s Guidelines for Exercise Testing and Prescription equation for energy expenditure (ml/kg/min O2) during leg cycling was used: *Resting component (3.5) + Horizontal component (3.5) + ((1.8* × *calculated max workload)* ÷ *body mass(kg)*. This is a standard procedure used when performing submaximal exercise testing (a notable example would be the YMCA submaximal cycle ergometer test; Fitchett, 1985; Beekley et al., 2004).

### Psychological and Cognitive Assessments

Before and after the 3-month intervention, participants took a series of neuropsychological tasks and self-reported questionnaires at home, at least 2–4 h but no longer than 7 days after their last exercise session. Neuropsychological tasks were administered *via* the lab’s website^3^ designed to present all cognitive tasks as well as store participant data. Self-reported questionnaires were administered *via* Google Forms. Participants were asked to refrain from drinking alcohol or using other illicit substances both before and during these assessments.

### Self-Report Questionnaires to Assess Psychological State

Participants were instructed to complete a series of validated and reliable self-report questionnaires to assess their affective state, eating attitudes, body image, and exercise motivation.

#### Affective State

The Beck Anxiety Inventory (BAI) and State Trait Anxiety Inventory (STAI) were used to assess anxiety. The BAI consists of 21 items scored on a 4-point Likert scale, and items are summed for a total score with higher scores reflecting greater anxiety (Beck et al., 1988a). The STAI consists of 40 items scored on a 4-point Likert scale, and items are summed for a total score with higher scores reflecting greater anxiety (Spielberger, 1983). The Beck Depression Inventory (BDI) was used to assess symptoms of depression (Beck et al., 1988b). The BDI consists of 21 items scored on a 4-point Likert scale, and items are summed for a total score with higher scores reflecting greater depressive symptoms. The Positive and Negative Affect Schedule (PANAS) was used to assess both positive affect and negative affect (Watson and Clark, 1999). The PANAS consists of 60 items to assess six subscales of positive affect including general positive affect, joviality, self-assurance, attentiveness, serenity, and surprise as well as seven subscales of negative affect including general negative affect, fear, sadness, guilt, hostility, shyness, and fatigue. Items are scored on a 5-point Likert scale, and items are summed to calculate a score for each subscale. The Perceived Stress Scale (PSS) was used to assess stress (Cohen et al., 1983). The PSS consists of 10 items scored on a 5-point Likert scale, and items are summed for a total score with higher scores reflecting greater stress. The Ruminative Response Scale (RRS) was used to assess rumination (Treynor et al., 2003). The RSS consists of 22 items scored on a 4-point Likert scale, and items are summed to calculate a total score.

#### Eating Attitudes and Body Image

The Eating Disorder Examination Questionnaire (EDE-Q) and the Eating Attitudes Test (EAT) were used to assess disordered eating behavior. The EDE-Q consists of 28 items including 22 items scored on a 7-point Likert scale assessing restraint, eating concern, shape concern, and weight concern, and six free-response items regarding frequency of behaviors (Berg et al., 2012). Restraint, eating concern, shape concern, and weight concern scores are averaged for a global score with higher scores reflecting more disordered eating. The EAT consists of 26 items scored on a 6-point Likert scale, and items are summed for a total score with higher scores reflecting more disordered eating (Garner et al., 1982). The Body Attitudes Test (BAT) was used to assess body image (Probst et al., 1995). The BAT includes twenty items that are scored on a 6-point scale, and items are summed for a total score with higher scores indicating more negative body image.

#### Exercise Motivation

The Behavioral Regulation of Exercise Questionnaire (BREQ-2) was used to assess self-determination for exercise (Markland and Tobin, 2004). The BREQ-2 consists of 19 items that are scored on a 5-point Likert scale, and averaged for five subscale scores including amotivation, external regulation, introjected regulation, identified regulation, and intrinsic regulation. The Relative Autonomy Index (RAI) is an index indicating the degree to which individuals are self-determined to exercise, and is calculated as a sum of weighted subscale scores (Ryan and Connell, 1989):


$$\begin{array}{l} \text{RAI= (Amotivation}*\text{-3)+(External Regulation}*\text{-2)+ Introjected Regulation}*\text{-}1)+\\ \text{(IdentifiedRegulation}*\text{2)+(Intrinsic Regulation}*\text{3)} \end{array}$$

### Tests to Assess Cognitive Function

#### Stroop Task

This classical test of executive function assessed both attention and the inhibition of cognitive interference (Stroop, 1935). Participants were presented with a series of colored words (i.e., red, green, blue, or yellow) and asked to indicate *via* button press the color of each word. The color of the word either matched (i.e., congruent; 50% of trials) or did not match (i.e., incongruent) the word itself (e.g., the word blue printed in blue ink is an example of a congruent trial whereas the word blue printed in red ink is an example of an incongruent trial). Three blocks with 48 trials per block were presented. Average percentage correct and reaction time were captured for congruent and incongruent trials. An interference score was calculated using the formula:


I=[(Total Correct Congruent) - (Total Correct Incongruent)]/[(Total Correct Congruent)+(Total Correct Incongruent)] * 100

adapted from the formula used by Valgimigli et al. (2010).

#### Eriksen Flanker Task

This test of executive function assessed both attention and response inhibition (Eriksen and Eriksen, 1974). Participants were presented with strings of seven letters, one central letter flanked by three letters on each side. Participants were instructed to focus on the center letter and look for one of four letters. The flankers either matched the central letter (i.e., congruent), did not match the central letter but was another of the three possible letters (i.e., incongruent), or was a different letter altogether (i.e., neutral). Participants were then instructed to press the arrow key that was associated with the central letter. Three blocks with 48 trials per block were presented. Average percentage correct and reaction time were captured for congruent, incongruent, and neutral trials.

#### N-Back Task

This test was used to test the participants’ working memory and measured their response times for each N-phase (Kirchner, 1958). Participants were presented with a series of letters for a total of four phases. The goal of this test was to correctly identify whether the current letter matched the target letter that was either zero, one, two, or three steps back in the series. The participants had to press “J” if the letter was a match or “F” if the letter was not a match. A key was provided at the bottom as a reminder. In the 0-Back phase, there was only one letter to match during the whole task, irrespective of letter capitalization. In the 1-Back phase, the participants had to remember the last letter presented to them immediately after the current one. In the 2-Back phase, the participants had to remember the letter that came two steps back before the current letter. Lastly, in the 3-Back phase, the participants had to remember the letter that came three steps back before the current letter. Each block consisted of 30 + n trials, with each block containing eight targets and three lures. Average percentage correct and reaction time were captured for 0-, 1-, 2-, and 3-back conditions.

#### Spatial Navigation Test

**Figure 1:**
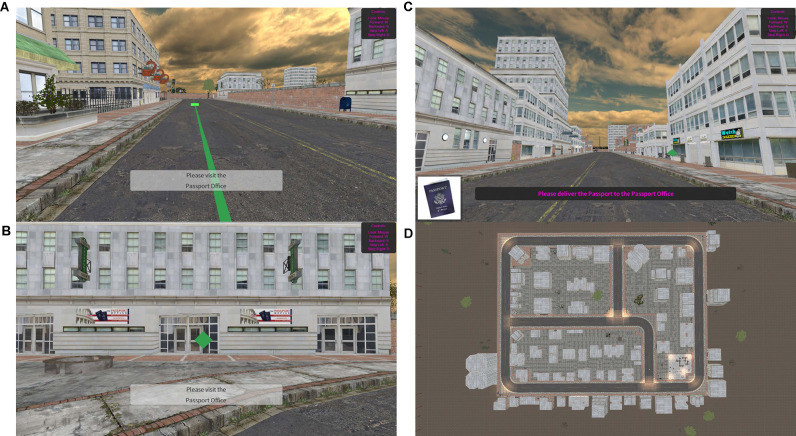
Spatial Navigation Tests. **(A)** Example scene in the encoding phase- green path with arrows leading to landmark. **(B)** Example scene in the remembering phase. **(C)** Example landmark with green diamond marker. **(D)** Aerial map of the game environment.

This test of hippocampal-dependent functioning assesses spatial navigation and episodic memory abilities. The spatial map utilized for this task and the task procedure were adapted from Miller et al. (2013). In this task, participants navigated their way through a virtual city by following a green path with arrows to locate specific landmarks (Figure 1A). Participants moved through the virtual city by moving forward (up arrow) or backward (down arrow) and turning right or left using the mouse. Participants were also able to look around the city (e.g., up to the sky or down to the ground) by using the mouse. These controls were presented to the participant on the screen as a reminder. Participants were required to visit five different landmarks and memorize where they were located. When the participant found the landmark, they were required to walk directly into a green diamond (Figure 1B). After this guided portion of the task, participants again completed the task without the guided arrows. Participants were asked to return to the landmarks in the same order they first visited them and deliver an item to each location (Figure 1C). An aerial view of the entire map is presented in Figure 1D. The time to find each location was captured for each portion of the task (i.e., encoding and remembering phase). Total time of task (end time − start time) and average seek duration for the encoding and remembering phase of the task were calculated. To assess episodic memory, participants were asked to freely recall (by typing words into boxes) all the landmarks they visited in order as well as the items they delivered to each landmark. Place score was calculated as the number of landmarks correctly recalled. Order score was calculated as the number of landmarks recalled in the correct position in the sequence. Item score was calculated as the number of items correctly recalled. Association score was calculated as the number of correct landmark and item pairings. Episodic memory score was calculated as a sum of the Place, Order, Item, and Association scores.

#### Mnemonic Similarity Test

This test of hippocampal- and extra-hippocampal-dependent functioning assesses recognition memory and lure discrimination, two aspects of memory (Stark et al., 2013). First, participants were presented with a series of images and asked to determine whether these items belonged indoors (button press “I”) or outdoors (button press “O”). This portion of the task ensured that participants were paying attention to the stimuli displayed on the screen. During the second, surprise portion of the task, participants were shown an additional 96 images and asked to identify whether the images were old (button press “F”), similar (space bar), or new (button press “J”); each category was presented $\frac{1}{3}$ of the time. Old images were those presented in the first set, similar images were those that were similar to the ones presented in the first set, and new images were those not presented in the first set. A key was provided at the bottom of the screen as a reminder for the appropriate button press. Participant’s response times for all trials were also recorded. Recognition memory performance was calculated as “old” responses to old images minus “old” responses to new images. Mnemonic similarity performance (i.e., Lure Discrimination Index) was calculated as “similar” responses to similar images minus “similar” responses to new images.

#### Statistical Analysis

A Repeated Measures Analysis of Variance (ANOVA) within-between groups was used to assess differences across time (pre- vs. *post-test*) and between groups (controls vs. increasers). A Pearson’s Product-Moment Correlation was used to assess relationships between the total number of classes and the change in each neurobehavioral measure as well as the change in estimated VO_2_ max and the change in each neurobehavioral measure. An alpha value of 0.05 was utilized to determine statistical significance. IBM SPSS Statistics Version 26 was utilized for all statistical analyses.

## Results

### Demographics

At baseline, there was no difference between groups in age (*t*_(78)_ = −0.384, *p* = 0.702), gender (χ^2^_(1)_ = 0.450, *p* = 0.502), or education (χ^2^_(3)_ = 6.969, *p* = 0.073; Table 1).

**Table 1:** Baseline demographic characteristics.

	**Control**	**Exercise**	**t/X2**	***p***
**N**	41	39		
**Age**	30.46 (4.92)	30.97 (6.85)	−0.384	0.702
**Gender**			0.450	0.502
% Female	82.9	76.9		
% Male	17.1	23.1		
**Education**			6.969	0.073
% High school	4.9	0		
% Some college	2.4	17.9		
% College degree	61	53.8		
% Advanced degree	31.7	28.2		

*Age presented as Mean (SD). All other variables presented as percentages*.

### Total Class Sessions

**Figure 2:**
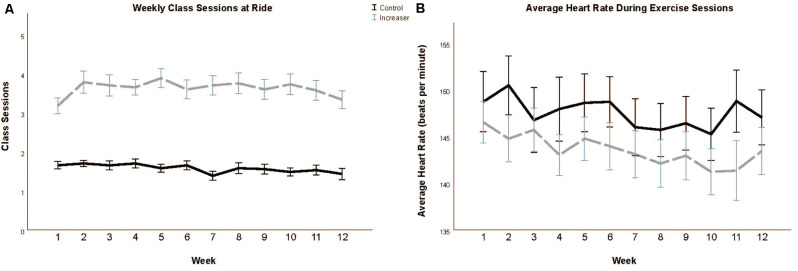
Total class sessions **(A)** and average heart rate during class sessions **(B)** during the 12-week intervention period. Data presented as means and standard errors.

The experimental group (47.87 ± 2.24) engaged in significantly more total cycling workouts over the course of the intervention than the control group (20.73 ± 0.72; *t*_(45.76)_ = −11.554, *p* < 0.001; Figure 2A). Additionally, the experimental group engaged in significantly more workouts outside of the cycling studio than the intervention group (*t*_(54.320)_ = −3.586, *p* < 0.001). We additionally confirmed that even when including workouts outside of the cycling studio, the control group maintained their previous regimen of 1–2 exercise sessions per week (23.63 (±1.11).

### Heart Rate During Class Sessions

There was no significant difference in average heart rate during class sessions between the experimental (145.38 ± 1.92) and control groups (144.96 ± 2.10; *t*_(78)_ = −0.147, *p* = 0.883; Figure 2B).

### Body Mass and Fitness

There was no significant time nor time^*^group effect on body mass or BMI. There was a significant time effect on estimated VO_2_ max (*F*_(1, 57)_ = 18.809, *p* < 0.001), with both groups increasing in fitness over time (Table 2). Of note, the average aerobic capacity of the control group at baseline was 33.46 ml/kg/min oxygen consumption and the increaser group was 30.52 ml/kg/min, thus confirming that both groups were moderately fit at baseline (Graves et al., 2015).

**Table 2:** Body mass and fitness.

	**Control**		**Increaser**		**TIME**	TIME * GROUP
	**Before**	**After**	**Before**	**After**	***p* value (effect size)**	***p* value (effect size)**
VO_2_ max	33.46 (5.22)	35.62 (4.88)	30.52 (4.56)	33.69 (5.65)	<0.001 (0.248)^*^	0.414 (0.012)
Body Mass (kg)	69.09 (13.57)	67.56 (11.98)	77.20 (19.16)	77.66 (18.80)	0.429 (0.009)	0.147 (0.031)
BMI	24.88 (4.61)	24.32 (3.93)	26.76 (5.74)	26.91 (5.54)	0.406 (0.010)	0.154 (0.030)

Data presented as Mean (SD). ^*^p < 0.05.

### Psychological Measures

#### Affective State

**Figure 3:**
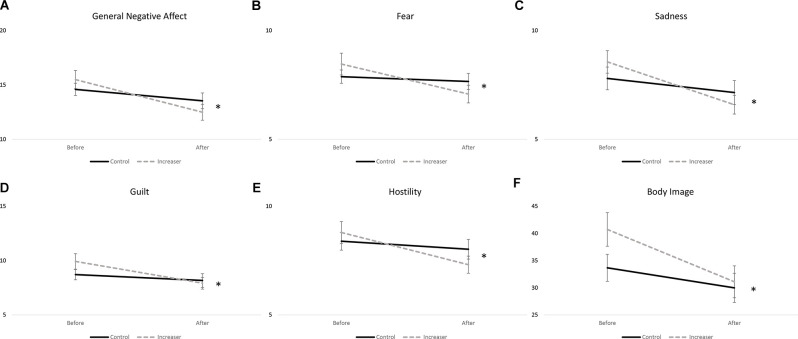
Significant time by group effects for **(A)** general negative affect; **(B)** fear; **(C)** sadness; **(D)** guilt; **(E)** hostility; and **(F)** body image. Data presented as mean ± SEM. ^*^p < 0.05.

A significant time^*^group interaction was found for general negative affect (*F*_(1, 78)_ = 4.667, *p* = 0.034), fear (*F*_(1, 78)_ = 4.873, *p* = 0.030), sadness (*F*_(1, 78)_ = 3.992, *p* = 0.049), guilt (*F*_(1, 78)_ = 4.152, *p* = 0.045), and hostility (*F*_(1, 78)_ = 5.367, *p* = 0.023) subscales of the PANAS (Figures 3A–E). Increasers had a significant decrease in general negative affect (*F*_(1, 38)_ = 29.772, *p* < 0.001), while controls had a non-significant decrease (*F*_(1, 40)_ = 2.189, *p* = 0.147). Increasers had a significant decrease in fear (*F*_(1, 38)_ = 13.980, *p* < 0.001), while controls had a non-significant decrease (*F*_(1, 40)_ = 0.342, *p* = 0.562). Increasers had a significant decrease in sadness (*F*_(1, 38)_ = 28.462, *p* < 0.001), while controls had a non-significant decrease (*F*_(1, 40)_ = 1.503, *p* = 0.227). Increasers had a significant decrease in guilt (*F*_(1, 38)_ = 21.794, *p* < 0.001), while controls had a non-significant decrease (*F*_(1, 40)_ = 0.887, *p* = 0.352). Increasers had a significant decrease in hostility (*F*_(1, 38)_ = 21.601, *p* < 0.001), while controls had a non-significant decrease (*F*_(1, 40)_ = 1.028, *p* = 0.317). Additionally, a significant time effect was found for all affective state measures except for shyness, with both groups showing improvements over time (Table 3).

**Table 3:** Affective state measures.

	**Control (*N* = 41)**		**Increaser (*N* = 39)**		**TIME**	TIME * GROUP
	**Before**	**After**	**Before**	**After**	***p* value (effect size)**	***p* value (effect size)**
Beck Anxiety Inventory	9.49 (7.45)	5.78 (7.05)	10.92 (7.91)	4.72 (6.61)	<0.001 (0.341)^*^	0.114 (0.032)
State Trait Anxiety Inventory	74.37 (15.86)	70.12 (20.18)	72.41 (19.08)	62.59 (18.67)	<0.001 (0.150)^*^	0.145 (0.027)
Beck Depression Inventory	8.83 (5.44)	5.98 (7.72)	9.72 (8.14)	4.82‘(6.27)	<0.001 (0.245)^*^	0.189 (0.022)
General Positive Affect	31.24 (6.91)	34.12 (7.17)	31.82 (6.92)	36.72 (7.92)	<0.001 (0.219)^*^	0.229 (0.019)
Joviality	25.85 (5.94)	27.85 (5.61)	26.44 (5.71)	30.23 (6.54)	<0.001 (0.169)^*^	0.221 (0.019)
Self Assuance	16.05 (3.71)	18.15 (4.18)	15.64 (4.59)	18.64 (4.97)	<0.001 (0.258)^*^	0.360 (0.011)
Attentiveness	11.88 (2.79)	13.37 (3.46)	12.62 (2.96)	14.23 (3.37)	<0.001 (0.180)^*^	0.865 (0.000)
Serenity	8.29 (2.62)	9.46 (2.16)	9.67 (2.37)	10.49 (2.80)	0.001 (0.129)^*^	0.552 (0.005)
Surprise	5.68 (1.95)	5.95 (2.38)	5.51 (2.04)	6.33 (2.26)	0.031 (0.058)^*^	0.269 (0.016)
General Negative Affect	14.59 (3.59)	13.54 (4.65)	15.49 (5.26)	12.49 (4.59)	<0.001 (0.205)^*^	0.034 (0.056)^*^
Fear	7.88 (1.96)	7.66 (2.37)	8.46 (3.13)	7.08 (2.54)	0.003 (0.106)^*^	0.030 (0.059)^*^
Sadness	7.80 (3.35)	7.15 (3.54)	8.56 (3.24)	6.59 (2.68)	<0.001 (0.170)^*^	0.049 (0.049)^*^
Guilt	8.71 (3.04)	8.17 (4.02)	9.92 (4.45)	7.92 (3.40)	<0.001 (0.138)^*^	0.045 (0.051)^*^
Hostility	8.39 (2.63)	8.02 (2.89)	8.79 (3.16)	7.31 (2.45)	<0.001 (0.158)^*^	0.023 (0.064)^*^
Shyness	5.95 (1.94)	5.56 (2.03)	6.26 (2.41)	5.85 (2.40)	0.054 (0.047)	0.961 (0.000)
Fatigue	9.83 (3.17)	7.66 (2.97)	9.74 (3.25)	7.15 (2.91)	<0.001 (0.307)^*^	0.606 (0.003)
Perceived Stress	14.98 (6.64)	13.05 (7.40)	14.85 (6.71)	11.56 (7.45)	<0.001 (0.160)^*^	0.319 (0.013)
Rumination	37.56 (10.57)	34.46 (13.35)	39.08 (12.21)	31.23 (10.28)	<0.001 (0.186)^*^	0.071 (0.041)

Data presented as Mean (SD). ^*^p < 0.05.

#### Eating Attitudes and Body Image

A significant time^*^group interaction was found for body image (*F*_(1, 78)_ = 5.019, *p* = 0.028; Figure 3F). Both groups showed significant improvements in body image, with increasers (*F*_(1, 38)_ = 17.686, *p* < 0.001) showing greater improvements than controls (*F*_(1, 40)_ = 6.985, *p* = 0.012). Additionally, a significant time effect was seen for disordered eating as measured by the EDE-Q (*F*_(1, 78)_ = 19.679, *p* < 0.001) and body image (*F*_(1, 78)_ = 25.220, *p* < 0.001), with both groups showing improvements over time (Table 4).

**Table 4:** Eating attitudes, body image, and exercise motivation.

	**Control (*N* = 41)**		**Increaser (*N* = 39)**		**TIME**	TIME * GROUP
	**Before**	**After**	**Before**	**After**	***p* value (effect size)**	***p* value (effect size)**
Eating Disorders Examination	2.06 (1.06)	1.77 (1.25)	2.15 (1.24)	1.70 (1.17)	<0.001 (0.201)^*^	0.320 (0.013)
Eating Attitudes Test	9.24 (7.85)	10.12 (9.89)	9.33 (6.87)	9.05 (8.14)	0.687 (0.002)	0.433 (0.008)
Body Image	33.66 (15.90)	29.98 (17.22)	40.72 (19.31)	31.10 (18.26)	<0.001 (0.244)^*^	0.028 (0.060)^*^
Exercise Motivation (RAI)	11.50 (4.75)	11.86 (5.36)	11.18 (4.89)	12.71 (4.05)	0.003 (0.108)^*^	0.063 (0.044)

Data presented as Mean (SD). ^*^p < 0.05.

#### Exercise Motivation

A significant time effect was seen for exercise motivation (*F*_(1, 78)_ = 9.480, *p* = 0.003) with both groups showing increased self-determination for exercise (Table 4).

### Cognitive Measures

#### Stroop Task

A significant time effect was found for percent correct on congruent (*F*_(1, 67)_ = 4.137, *p* = 0.046) and incongruent (*F*_(1, 67)_ = 5.100, *p* = 0.027) trials as well as reaction time on congruent correct (*F*_(1, 67)_ = 38.585, *p* < 0.001) and incongruent correct (*F*_(1, 67)_ = 13.575, *p* < 0.001) trials (Supplementary Table 1). No significant time^*^group effects were observed.

#### Eriksen Flanker Task

A significant time effect was found for reaction time on incongruent correct (*F*_(1, 68)_ = 5.465, *p* = 0.022) and neutral correct trials (*F*_(1, 68)_ = 5.634, *p* = 0.020) with both groups decreasing their reaction times over time (Supplementary Table 2). No other time effects nor time^*^group effects were observed.

#### N-Back Task

A significant time effect was found for target percent correct on N0 (*F*_(1, 68)_ = 12.550, *p* < 0.001) trials as well as for reaction time on correct N2 trials (*F*_(1, 62)_ = 6.684, *p* = 0.012; Supplementary Table 3). No other time effects nor time^*^group effects were observed.

#### Spatial Navigation Test

Due to non-completion of this task, *n* = 11 participants are missing from final analysis. A significant time effect was seen for Order Score (*F*_(1, 67)_ = 9.649, *p* = 0.003), Association Score (*F*_(1, 67)_ = 10.593, *p* = 0.002), and Episodic Memory Score (*F*_(1, 67)_ = 4.233, *p* = 0.044), with both groups increasing their score over time. No other significant time nor time^*^group effects were observed for any variables (Table 5).

**Table 5:** Spatial navigation test.

	**Control (*N* = 35)**		**Increaser (*N* = 34)**		**TIME**	TIME * GROUP
	**Before**	**After**	**Before**	**After**	***p* value (effect size)**	***p* value (effect size)**
Average Seek Duration	69.63 (42.04)	77.01 (65.02)	93.87 (72.47)	74.74 (43.60)	0.462 (0.008)	0.100 (0.040)
Total Time	274.95 (169.97)	300.33 (294.42)	346.73 (295.89)	303.40 (197.50)	0.804 (0.001)	0.342 (0.013)
Place Score	4.31 (0.80)	4.11 (1.32)	4.38 (0.99)	4.26 (1.36)	0.356 (0.013)	0.810 (0.001)
Item Score	3.66 (1.03)	3.60 (1.50)	3.53 (0.96)	3.82 (1.62)	0.538 (0.006)	0.362 (0.012)
Order Score	3.46 (1.56)	4.23 (1.33)	3.85 (1.46)	4.38 (1.23)	0.003 (0.126)^*^	0.565 (0.005)
Association Score	3.49 (1.22)	4.03 (1.15)	3.65 (0.81)	4.29 (1.34)	0.002 (0.137)^*^	0.777 (0.001)
Episodic Memory Score	14.91 (4.02)	15.97 (4.37)	15.41 (3.14)	16.76 (4.38)	0.044 (0.059)^*^	0.801 (0.001)

Data presented as Mean (SD). ^*^p < 0.05.

#### Mnemonic Similarity Task

Due to non-completion of this task, *n* = 10 participants are missing from final analysis. A significant time effect was seen for the mnemonic similarity performance as measured by the Lure Discrimination Index (*F*_(1, 68)_ = 12.410, *p* < 0.001), with both groups increasing their score over time. No significant time^*^group effects were observed (Table 6).

**Table 6:** Mnemonic similarity task.

	**Control (*N* = 35)**		**Increaser (*N* = 35)**		**TIME**	TIME * GROUP
	**Before**	**After**	**Before**	**After**	***p* value (effect size)**	***p* value (effect size)**
Lure Discrimination Index	0.38 (0.23)	0.44 (0.24)	0.34 (0.21)	0.48 (0.26)	<0.001 (0.154)^*^	0.169 (0.028)
Recognition Score	0.83 (0.17)	0.83 (0.16)	0.81 (0.24)	0.82 (0.15)	0.802 (0.001)	0.802 (0.001)

Data presented as Mean (SD). ^*^p < 0.05.

### Correlational Analyses Between Total Number of Cycling Workouts and Change in Psychological and Cognitive Metrics

**Figure 4:**
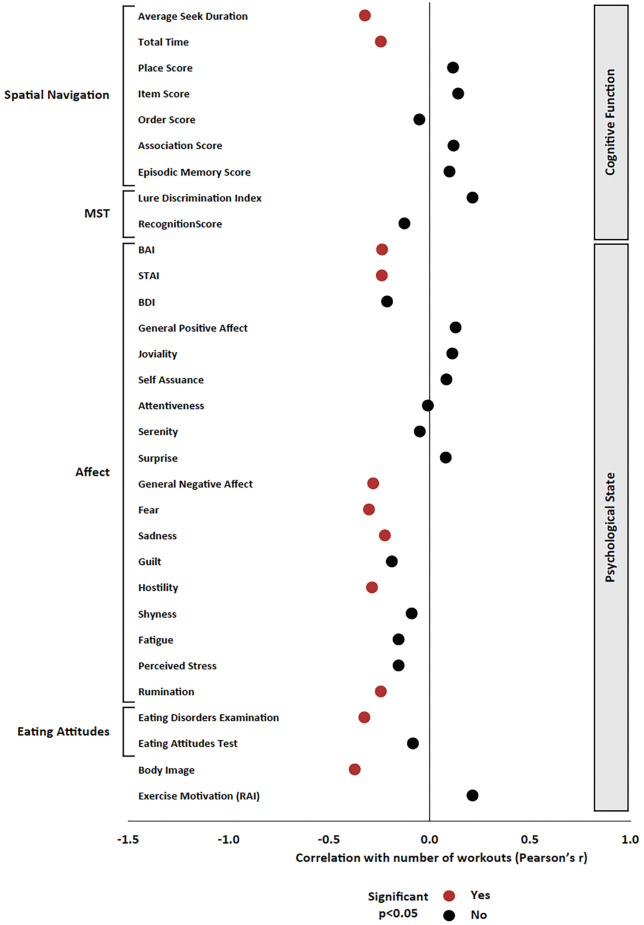
Pearson’s product-moment correlation between the total number of cycling workouts and all variables of interest. Pearson’s r is presented on the x axis, and significant effects (*p* < 0.05) are presented in red; non-significant effects are presented in black.

All correlational analyses included all participants, both in the control and experimental groups. In regard to the psychological measures, the total number of cycling workouts was significantly correlated with reduced anxiety (BAI *r* = −0.236, *p* = 0.035; STAI *r* = −0.237, *p* = 0.035), general negative affect (*r* = −0.280, *p* = 0.012), fear (*r* = −0.301, *p* = 0.007), sadness (*r* = −0.222, *p* = 0.048), hostility (*r* = −0.286, *p* = 0.010), rumination (*r* = −0.242, *p* = 0.030), and disordered eating as measured by the EDE-Q (*r* = −0.324, *p* = 0.003) as well as improved body image (*r* = −0.372, *p* = 0.001; Figure 4).

Regarding cognitive measures, the total number of cycling workouts was significantly correlated with the improvement in average seek duration (*r* = −0.321, *p* = 0.007) as well as the improvement in total time (*r* = −0.242, *p* = 0.045) in the Spatial Navigation Task (Figure 4). There were no significant correlations between the total number of cycling workouts and MST, Stroop, Eriksen Flanker, or N-back measures.

### Correlational Analyses Between Change in Fitness Level and Change in Psychological and Cognitive Metrics

**Figure 5:**
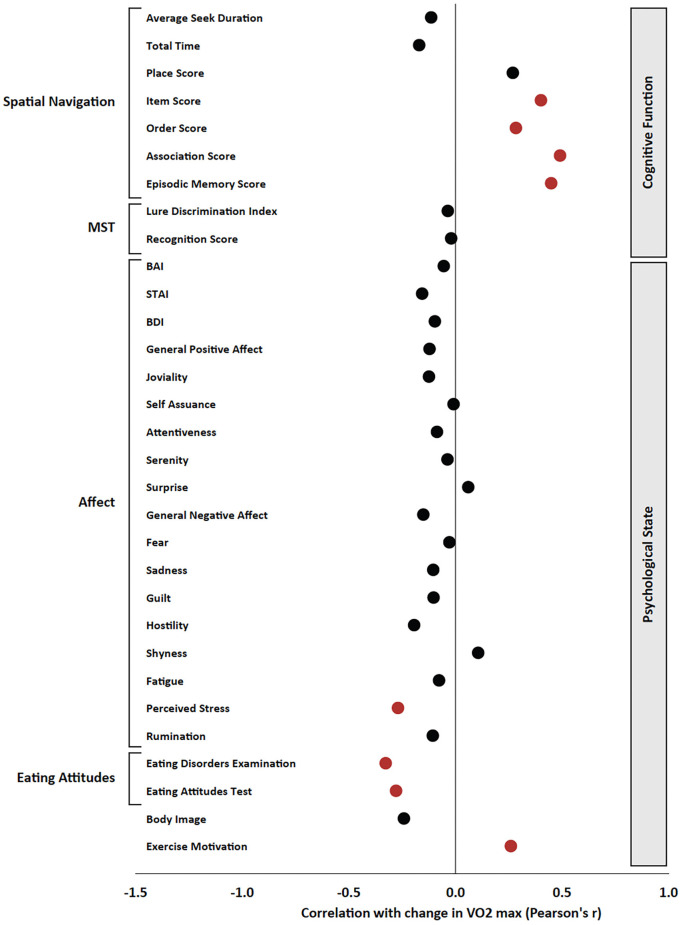
Pearson’s product-moment correlation between the change in estimated VO_2_ max and all variables of interest. Pearson’s r is presented on the x axis, and significant effects (*p* < 0.05) are presented in red; non-significant effects are presented in black.

Regarding psychological measures, increased estimated VO_2_ max was significantly correlated with decreased stress (*r* = −0.269, *p* = 0.039) and disordered eating (EDE-Q *r* = −0.327, *p* = 0.011; EAT *r* = −0.278, *p* = 0.033) as well as increased self-determination for exercise (*r* = 0.260, *p* = 0.047; Figure 5).

Regarding cognitive measures, increased estimated VO_2_ max was significantly correlated with improvements in the Item Score (*r* = 0.401, *p* = 0.004), Order Score (*r* = 0.284, *p* = 0.043), Association Score (*r* = 0.491, *p* < 0.001), and Episodic Memory Score (*r* = 0.449, *p* < 0.001) in the Spatial Navigation Task (Figure 5). Additionally, increased estimated VO_2_ max was correlated with decreased percent correct on N3 trials of the N-back task (*r* = −0.277, *p* = 0.049). There were no significant correlations between changes in estimated VO_2_ max and changes in MST, Stroop, or Eriksen Flanker measures.

## Discussion

The present study examined the effects of increasing the volume of an aerobic exercise program compared to standard exercise volume on affective state and cognitive performance among a group of moderately fit individuals. Using this randomized-control design, we found that compared to maintaining a moderate exercise regimen, increasing exercise significantly decreased levels of general negative affect, including fear, sadness, guilt, and hostility, as well as improved body image. Clearly, the strongest direct effect seen with aerobic exercise was on psychological state, but other findings emerged when looking at the data in a cross-sectional manner. Using correlational analysis across the entire population of the study, we found that the total number of workouts was significantly associated with improved spatial navigation abilities and body image as well as reduced anxiety, general negative affect, fear, sadness, hostility, rumination, and disordered eating. In addition, increases in fitness level were significantly associated with improved episodic memory and exercise motivation as well as decreased stress and disordered eating. Our findings (summary in Table 7) are some of the first to indicate that in middle-aged adults, continuing to increase exercise levels in an already ongoing fitness regimen has additional benefits for both psychological and cognitive health. These findings have important implications for healthy aging, especially as it relates to affective state and cognitive functioning. These findings in humans are consistent with exercise studies in rodents showing improved hippocampal functions associated with increased levels of exercise (van Praag et al., 1999a, b; Voss et al., 2013).

**Table 7:** Summary of the causal and correlational effects of chronic aerobic exercise in middle-aged adults. Causal effects refer to time^*^group effects.

	**Causal effects**	**Relationship to # of workouts**	**Relationship to fitness changes**
**Mood effects**			
General negative affect	X	X	
Fear	X	X	
Sadness	X	X	
Guilt	X		
Hostility		X	
Rumination		X	
Anxiety		X	
Stress			X
Anxiety		X	
Body image	X	X	
Disordered eating		X	X
**Motivation effects**			
Exercise motivation			X
**Cognitive effects**			
Episodic memory			X
Spatial memory		X	

### The Effects of Chronic Increases in Exercise on Psychological State

Following 12 weeks of aerobic exercise training, marked decreases in several negative mood indicators were seen including decreases in hostility, guilt, sadness, fear, and general negative affect, with the experimental group showing greater decreases than controls. A large body of work has shown that both acute and chronic exercise is beneficial at improving mood, including both increases in positive affect and decreases in negative affect, especially anxiety and depression (Cramer et al., 1991; Arent et al., 2000; Hoffman and Hoffman, 2008; Basso and Suzuki, 2017; Bonham et al., 2018; Aparicio et al., 2021). Here, we show that increasing aerobic exercise in middle-aged individuals with a previous exercise regimen can confer additional affective state improvements beyond maintaining an exercise regimen. Though the majority of studies have focused on younger or older populations (Reed and Ones, 2006; Chang et al., 2012; Hogan et al., 2013) and newer research has focused on middle-aged populations (Chen et al., 2019; Quinlan et al., 2021), our work is some of the first longitudinal exercise research in middle-aged adults. These beneficial effects on affective state are in part thought to be due to exercise-induced increases in neurotrophins and neuromodulators including dopamine, serotonin, norepinephrine, endocannabinoids, and endogenous opioids (Dietrich and McDaniel, 2004; Fuss and Gass, 2010; Lin and Kuo, 2013; Siebers et al., 2021). Additionally, other research has shown that affective-state changes may result from the fact that exercise produces a stress-resistant brain, having potent effects on the sympathetic-adrenomedullary and hypothalamus-pituitary-adrenal (HPA) axes (Greenwood and Fleshner, 2008; Fleshner et al., 2011). Future research is warranted to determine the structural and physiological brain changes associated with these exercise-induced mood-state changes and how long after exercise cessation they persist.

In addition to the decreased negative mood parameters, we found decreases in negative body image after 12 weeks of training, with the experimental group showing greater decreases than controls. Interestingly, both groups demonstrated decreases in negative body image despite lack of change in either BMI or eating attitudes. Regardless, those who increased their exercise regimen demonstrated significantly greater reductions in negative body image compared to the control group. Other research has shown that exercise interventions improve body image in a range of age groups across the lifespan (Hausenblas and Fallon, 2006; Campbell and Hausenblas, 2009). While BMI was assessed before and after 12 weeks, there were no measures of body composition nor of body circumferences. Thus, there may have been redistributions of weight from fat mass to fat-free mass, or changes in waist circumferences that influenced the improved body attitudes. Future research may seek to disentangle the changes in negative body image that are due to the act of performing exercise and the potential anthropometric and body compositional changes that result from that exercise.

We also found that greater engagement in exercise and improved cardiorespiratory fitness improved mood, eating motivation, exercise motivation, and body image. This is one of the first studies to show exercise frequency-related improvements in affect, motivation, and body image in healthy, active adults.

In line with our findings, previous work has demonstrated that exercise leads to decreased anxiety symptoms for individuals with anxiety disorders as well as healthy individuals (Mochcovitch et al., 2016; Stubbs et al., 2017). Additionally, the association between number of workouts or fitness gains and reduction in rumination, general negative affect, sadness, and hostility is consistent with previous findings indicating that increased exercise reduces the risk of depression (Hassmén et al., 2000; Mammen and Faulkner, 2013). Importantly, our work and others have shown that exercise engagement may reduce negative mood state even in the absence of changes in cardiorespiratory fitness (Olson et al., 2017). Research additionally shows that exercise or cardiorespiratory fitness may be related to improved physiological stress reactivity and reduced psychological stress (Holmes and Roth, 1985; Aldana et al., 1996; Brockmann and Ross, 2020; Allesøe et al., 2021). In a large cross-sectional sample (*N* = 55,185), Allesøe et al. (2021) found that higher levels of self-reported physical activity and fitness were associated with lower levels of perceived stress. Our current study also found that improved fitness, measured through an objective exercise test, is related to decreased perceived stress. Collectively, these findings suggest that exercise engagement and improved fitness lead to improvements in a range of negative mood measures.

Although some studies have found that engagement in a regular exercise regimen leads to improvements in exercise motivation and body image (Pearson and Hall, 2013), the relationships between these findings and either number of workouts or gains in fitness have not previously been reported. Additionally, some studies have suggested that the effect of exercise on body image may be moderated by exercise motivations indicating that there is a complex relationship among psychological outcomes affected by exercise (Lepage and Crowther, 2010). Although there is limited research regarding the relationship between physical fitness and eating motivations, one study found that adolescents with lower levels of fitness had a greater risk of developing eating disorders (Veses et al., 2014). As much of the previous research in this area has focused on the relationship between compulsive exercise and eating disorders, this is the first interventional study to show a relationship between improvements in fitness and decreased disordered eating in a healthy population.

### The Effects of Chronic Increases in Exercise on Cognitive Functioning

Additionally, we found that greater engagement with exercise and improvements in cardiorespiratory fitness were significantly associated with improvements in spatial navigation and episodic memory abilities. That is, individuals who exercised more frequently and showed larger gains in fitness were more efficiently able to navigate to previously learned locations as well as remember information presented to them during this experience, tasks that are both dependent largely on the hippocampal formation. This is the first time that exercise has been shown to improve spatial navigation in a healthy adult population using a virtual maze task. A recent pilot study in 14 older adults (aged ≥ 60) found that 2 months of exergaming significantly improved spatial navigation as assessed by the immediate course performance maze time in the Floor Maze Test, a task of exocentric and allocentric navigation (Oliveira et al., 2020). Previous reports have also found cross-sectional evidence in adolescents that enhanced cardiorespiratory fitness levels are associated with increased hippocampal volume, which was subsequently associated with spatial learning on a Virtual Water Morris Maze (Herting and Nagel, 2012; Prathap et al., 2021). Other cross-sectional work has shown significant positive relationships between physical activity level (e.g., total step count and step rate) and episodic memory ability in older adults (Hayes et al., 2015). Additionally, acute exercise has been shown to have a significant positive effect on episodic memory (Sng et al., 2018; Johnson and Loprinzi, 2019; Loprinzi et al., 2019). We add to this literature by showing for the first time that enhancing physical activity and fitness in middle-aged adults improves both spatial and episodic memory abilities. This is consistent with extant research in rodents showing that exercise improves hippocampal-dependent functioning, especially spatial navigation (Voss et al., 2013).

Previous work in both human and animal literature has identified that this effect is due to exercise-induced changes in the hippocampus, a critical structure for learning and memory. A pivotal study in older adults found that a one-year intervention of aerobic walking increased the volume of the anterior hippocampus, which included the subiculum, CA1, and dentate gyrus, the seat of exercise-induced neurogenesis (Erickson et al., 2011). This increase in bilateral hippocampal volume was positively associated with the change in VO_2_ max, serum BDNF levels, and memory performance (using the dots fixation task) — though the aerobic exercise intervention did not have a significant effect on spatial memory performance itself. Another hallmark study found that chronic exercise increases dentate gyrus neurogenesis and cerebral blood volume (CBV) in rodents in parallel to dentate gyrus CBV in humans (ranging from 21 to 45 years of age), which correlated to increases in both VO_2_ max and learning on the Rey Auditory Verbal Learning Task (Pereira et al., 2007). Additionally, an extant body of rodent research has shown that voluntary wheel running and forced treadmill running improves spatial navigation through tasks such as the Morris Water Maze, Y-maze, T-maze, and radial arm maze as well as other tasks dependent on the hippocampus such as contextual fear conditioning, passive avoidance learning, novel object recognition, and pattern separation (Fordyce and Farrar, 1991; Van Praag et al., 2005; O’Callaghan et al., 2007; Chen et al., 2008; van Praag, 2008; Creer et al., 2010; Falls et al., 2010). The behavioral effects appear dependent on exercise-induced enhancements in hippocampal BDNF levels, neurogenesis, long-term potentiation, and functional integration into the existing hippocampal network (Neeper et al., 1995; van Praag et al., 1999a, b; Kobilo et al., 2011; Vivar et al., 2016; Voss et al., 2019).

This collection of research in both the animal and human literature suggests that exercise-induced improvements in spatial navigation and episodic memory are dependent upon both structural and physiological changes at the level of the hippocampus. We add to this literature in humans by demonstrating that greater engagement with exercise can enhance hippocampal abilities into middle age, which has significant implications for aging as detriment in these cognitive abilities emerge with age-related atrophy of hippocampal structures (Ramanoël et al., 2019).

In the current study, we also found no effects of increased exercise engagement on prefrontal cortex-dependent function as measured by the Stroop, Eriksen Flanker, and N-back tasks. These findings come in contrast to our work that shows an acute bout of aerobic exercise in adults of a similar age range improves prefrontal cortex functioning (Basso et al., 2015). We speculate that while acute exercise, with its numerous neural mechanisms of action (especially at prefrontal cortical sites; Basso and Suzuki, 2017), may acutely improve executive function, this level of chronic exercise is not stringent enough to induce baseline executive function improvements. We further hypothesize that the present null finding may be due to a ceiling effect on task performance as percentage correct in each of these tasks was at or near 100%. Previous work in this area has shown that aerobic exercise training significantly improves prefrontal cortex-dependent functioning, specifically using tasks including the Stroop, Eriksen Flanker, and N-Back Task (Dustman et al., 1984; Colcombe et al., 2004; Hansen et al., 2004; Smiley-Oyen et al., 2008; Stroth et al., 2010; Coetsee and Terblanche, 2017; Ludyga et al., 2018; Amatriain-Fernández et al., 2021). The majority of these studies were conducted in older adults (Dustman et al., 1984; Colcombe et al., 2004; Smiley-Oyen et al., 2008; Coetsee and Terblanche, 2017), while some were conducted in children or adolescents (Ludyga et al., 2018; Amatriain-Fernández et al., 2021). Other work in this area has produced null findings (Madden et al., 1989; Panton et al., 1990; Blumenthal et al., 1991; Hassmén et al., 1992; Hill et al., 1993; Dustman et al., 1994), with this lack of effect being attributed to methodological factors such as baseline levels of high cognitive functioning or short intervention periods that do not lead to fitness changes. Kramer et al. (1999), in fact, proposed a “selective improvement” hypothesis whereby aerobic exercise specifically acts on brain regions and cognitive processes that are sensitive to neurodegeneration, such as the prefrontal cortex and executive function. This hypothesis extends to the idea that aerobic exercise may only provide beneficial effects if: (1) enough cognitive decline is in place (i.e., there is room for cognitive improvement); or (2) the cognitive challenge is sufficient (i.e., tasks are challenging enough to show improvement). In fact, previous work in rodents and humans have indicated that the effects of exercise on cognition may be dependent on task difficulty (Creer et al., 2010; Déry et al., 2013; Heisz et al., 2017; Suwabe et al., 2017b). We suggest that there may be a “sweet spot” to examine the effects of exercise on cognitive function, whereby baseline levels of cognition demonstrate room for improvement (e.g., elderly or other clinical populations) or the task demands are challenging enough such that participants will be able to improve performance. Recently, studies in our lab have demonstrated that task difficulty can be modulated by decreasing the amount of time that participants are given the opportunity to respond to stimuli (e.g., decreasing stimuli presentation time from 1,500 ms to 1,000 ms). Future studies are warranted to systematically test the effects of exercise on prefrontal cortex tasks with various stimuli presentation times.

### Limitations and Future Directions

We acknowledge several limitations of the current study. First, our findings may be biased by the relatively high dropout rate (~38%) in the study. This dropout rate occurred most likely due to the remote nature of the study and despite several recruitment strategies including emails, text messages, and phone calls. Second, though we found extant correlational relationships between exercise sessions and cognitive and psychological measures, we found limited between groups effects on these outcomes—suggesting that our intervention and control groups may not have been distinct enough to show the effects of increased exercise engagement. For example, the experimental group attended on average, 4 weekly exercise sessions, which was on the low end of the assigned range of 4–7 weekly exercise sessions. Additionally, as all participants were confirmed as having a moderate exercise regimen for at least 3 months before the study began, they were all highly motivated to exercise. Most participants, including the control group, engaged in other forms of exercise (e.g., running, yoga, aerobic exercise classes) during the study period, and therefore, this additional amount of exercise may have contributed to mood, motivation, or cognitive effects. Further, the fact that participants were enrolled in an exercise study and assessed at two time points may have motivated them to exercise more. We also did not obtain a good estimate of workout intensity for all workouts completed outside of the cycling studio, which is another limitation. General physical activity levels were also not assessed; future studies may want to consider utilizing activity monitoring throughout the study period or self-reported questionnaires (e.g., International Physical Activity Questionnaire; Global Physical Activity Questionnaire). Alternatively, the lack of causational findings (i.e., lack of time^*^group effects) on hippocampal function may indicate that it may take a longer bout of exercise to cause hippocampal changes due to the involvement of stimulating the growth of new hippocampal neurons; 3 months of exercise may be too short of a time period for hippocampal cellular integration and functional improvement. Considering that previous reports of exercise-induced improvements in hippocampal function have been in young adults in response to high-intensity exercise regimens (Déry et al., 2013; Heisz et al., 2017), the age of our study population may contribute to the lack of time^*^group effects; moreover, higher exercise intensity may be needed to induce effects. Another limitation comes in the realm of the cardiorespiratory fitness test. Because the study happened remotely, we needed to rely on the participants themselves as well as staff at the fitness facility to help set up and start the equipment. We were not able to conduct a traditional VO_2_ max test and as such needed to calculate an estimate of aerobic capacity, and in some instances, technical issues arose with the test.

Considering the behavioral effects, future studies should investigate both the structural and functional changes associated with these improvements through neuroimaging techniques such as MRI and fMRI. We hypothesize that these behavioral improvements will be accompanied by increases in hippocampal volume as well as heightened functional activation during task engagement. Additionally, future research is warranted to investigate cellular and molecular mechanisms underlying this effect, especially for growth factors such as brain-derived neurotrophic factors (BDNF). Incorporating genetic testing for the BDNF gene may be an interesting avenue of research, specifically to determine whether individuals with different genetic variants (e.g., BDNF Val66Met polymorphism) may be more susceptible to these exercise-induced effects.

## Conclusions

In this long-term, randomized controlled study, we found that increasing exercise engagement was associated with improvements in hippocampal-dependent spatial memory, negative affective state, disordered eating, and body image in a population of moderately fit, middle-aged adults with an existing exercise regimen. Additionally, improved cardiorespiratory fitness was correlated with improvements in episodic memory, stress, and exercise motivation. These results suggest that among individuals with an existing exercise regimen, increasing the frequency of exercise provides further improvements in cognitive function, mood, and motivation. Our findings have clinical relevance and suggest that exercise may be one way to support healthy aging, especially for neurobehaviors that demonstrate age-related decline, including affective state and spatial learning and memory.

## Data Availability Staement

The raw data supporting the conclusions of this article will be made available by the authors, without undue reservation.

## Ethics Statement

The studies involving human participants were reviewed and approved by New York University Committee on Activities Involving Human Subjects. The patients/participants provided their written informed consent to participate in this study.

## Author Contributions

WS and JB conceptualized the study. ZP created all of the computerized neurocognitive tests for the study. JB, CO’B, and CC ran all study procedures. DO analyzed the fitness data. AM helped with data cleaning. JB and MS conducted all data analysis. JB, DO, and MS wrote the article and created all figures and tables. All authors contributed to the article and approved the submitted version.

## Conflict of Interest

The authors declare that the research was conducted in the absence of any commercial or financial relationships that could be construed as a potential conflict of interest.

## Publisher’s Note

All claims expressed in this article are solely those of the authors and do not necessarily represent those of their affiliated organizations, or those of the publisher, the editors and the reviewers. Any product that may be evaluated in this article, or claim that may be made by its manufacturer, is not guaranteed or endorsed by the publisher.

## Footnotes

^1^ https://www.ride-indoorcycling.com/

^2^ https://mio-labs.com/

^3^ www.suzukicognition.com

## Supplementary Material

Supplementary Figure S1 An increase in estimated VO_2_ max is significantly correlated with **(A)** a decrease in perceived stress level (PSS); **(B)** a decrease in disordered eating (EDE-Q); **(C)** a decrease in disordered eating (EAT); **(D)** an increase in exercise self-determination; and **(E)** an increase in episodic memory performance (data is similar for the subscales of item score, order score, and association score but not presented here for simplicity).

Supplementary Figure S2 Engagement in more cycling classes over the course of 12 weeks is significantly correlated with **(A)** a decrease in anxiety level (BAI); **(B)** a decrease in anxiety level (STAI); **(C)** a decrease in general negative affect (data is similar for the subscales of fear, sadness, and hostility but not presented here for simplicity); **(D)** a decrease in rumination; **(E)** a decreased in disordered eating (EDE-Q); **(F)** a decrease in negative body image; **(G)** a decrease in the average amount of time to find a location in the spatial navigation task; and **(H)** a decrease in the total time to find all locations in the spatial navigation task.
