# Supplementary figures and images for: Examining the Effect of Increased Aerobic Exercise in Moderately Fit Adults on Psychological State and Cognitive Function
Source: Front Hum Neurosci. 2022 Jul 12;16:833149. doi: 10.3389/fnhum.2022.833149 (PMC9317941; doi:10.3389/fnhum.2022.833149)

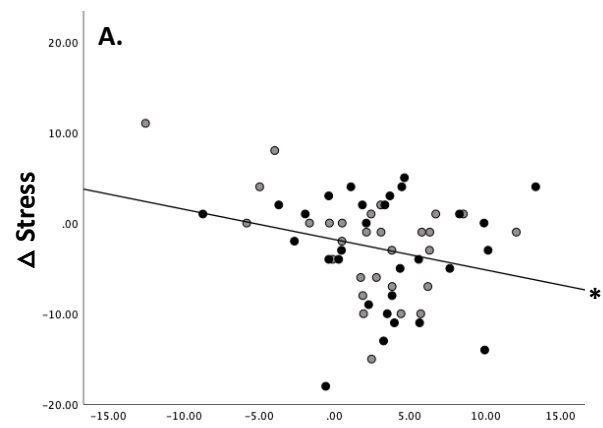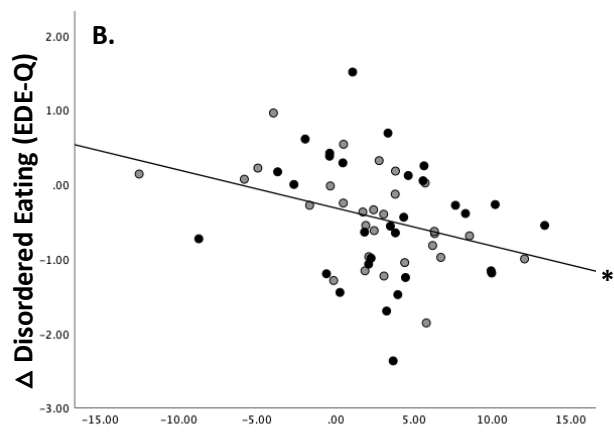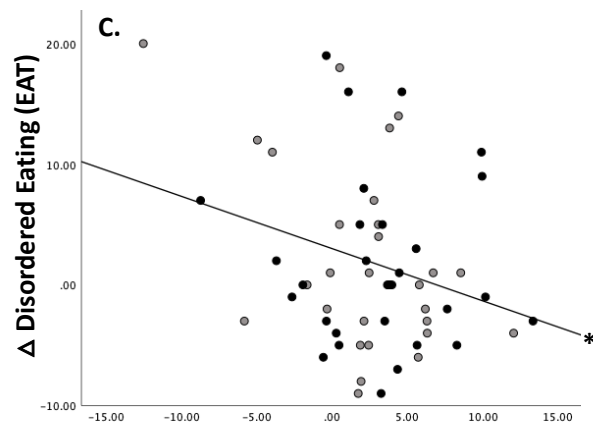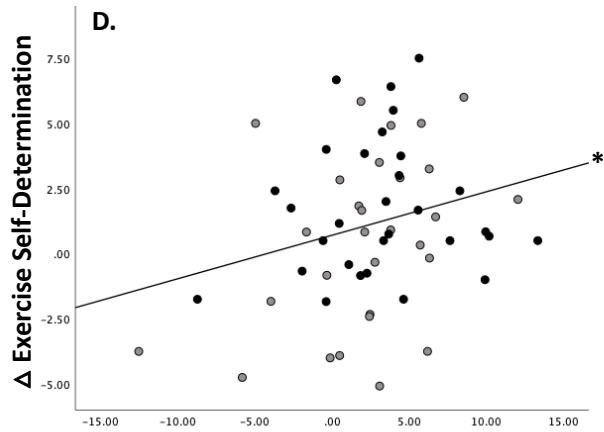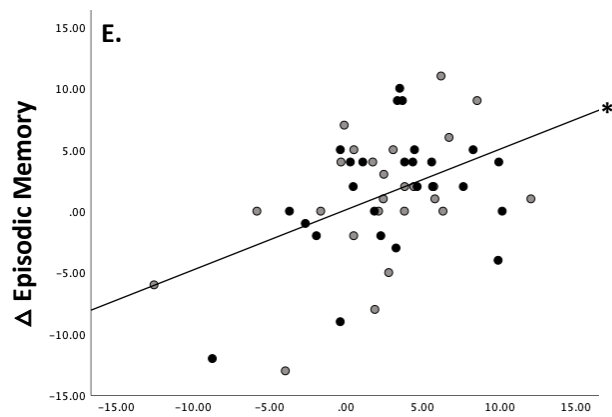

● Control  
● Increaser

Change in Estimated VO<sub>2</sub> Max

Supplement: Supplementary Figure S1 — An increase in estimated VO2 max is significantly correlated with (A) a decrease in perceived stress level (PSS); (B) a decrease in disordered eating (EDE-Q); (C) a decrease in disordered eating (EAT); (D) an increase in exercise self-determination; and (E) an increase in episodic memory performance (data is similar for the subscales of item score, order score, and association score but not presented here for simplicity). [file Data_Sheet_1.PDF]

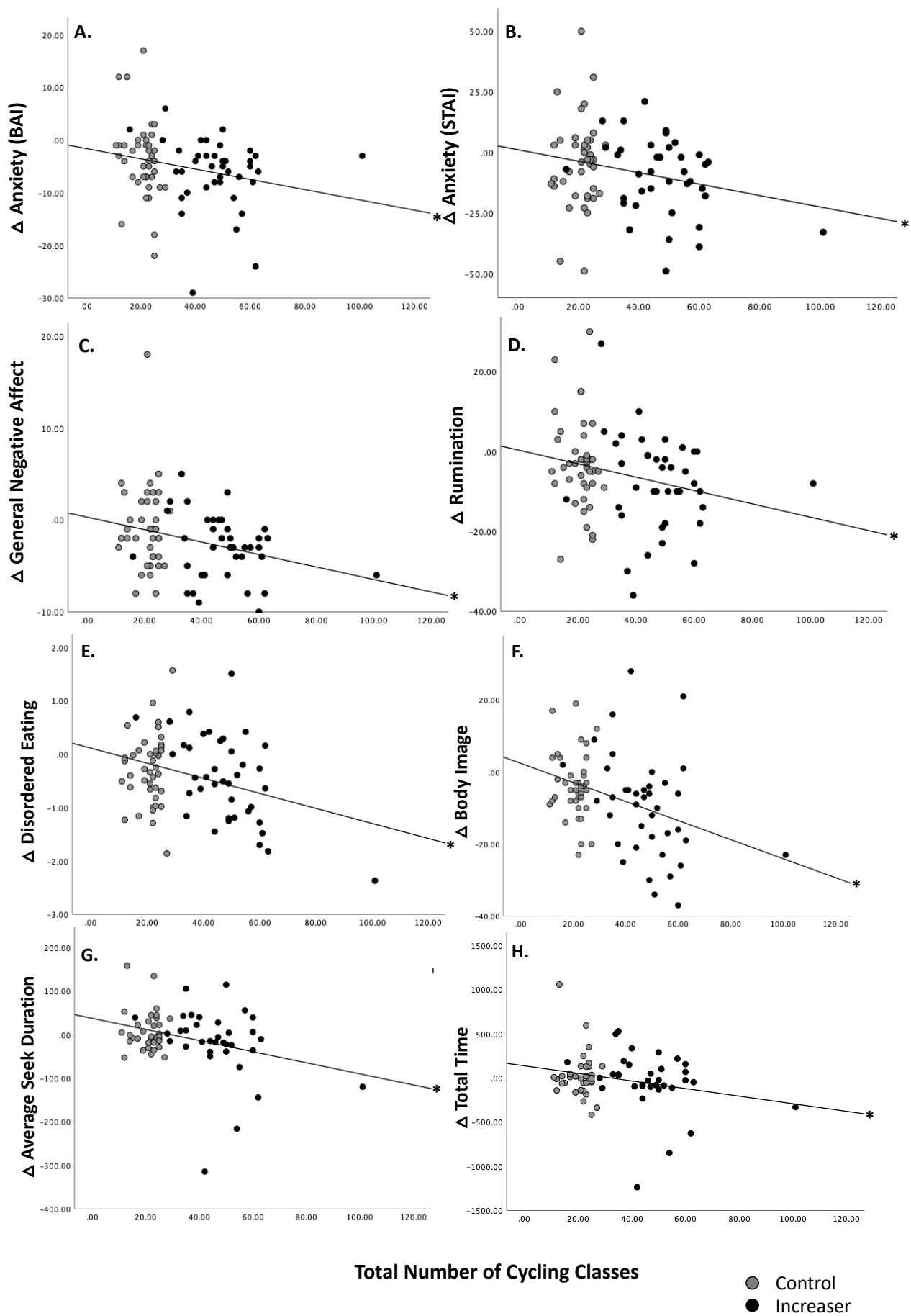

Supplement: Supplementary Figure S2 — Engagement in more cycling classes over the course of 12 weeks is significantly correlated with (A) a decrease in anxiety level (BAI); (B) a decrease in anxiety level (STAI); (C) a decrease in general negative affect (data is similar for the subscales of fear, sadness, and hostility but not presented here for simplicity); (D) a decrease in rumination; (E) a decreased in disordered eating (EDE-Q); (F) a decrease in negative body image; (G) a decrease in the average amount of time to find a location in the spatial navigation task; and (H) a decrease in the total time to find all locations in the spatial navigation task. [file Data_Sheet_2.PDF]
